# LifeWatchGreece Portal development: architecture, implementation and challenges for a biodiversity research e-infrastructure

**DOI:** 10.3897/BDJ.4.e9434

**Published:** 2016-11-01

**Authors:** Alexandros Gougousis, Nicolas Bailly

**Affiliations:** ‡ Hellenic Centre for Marine Research (HCMR), Gouves, Greece

**Keywords:** Biodiversity Information System, Research e-Infrastructure, Virtual Research Environment (VRE), Virtual Laboratories (vLab), e-service

## Abstract

**Background:**

Biodiversity data is characterized by its cross-disciplinary character, the extremely broad range of data types and structures, and the plethora of different data sources providing resources for the same piece of information in a heterogeneous way. Since the web inception two decades ago, there are multiple initiatives to connect, aggregate, share, and publish biodiversity data, and to establish data and work flows in order to analyze them. The European program LifeWatch aims at establishing a distributed network of nodes implementing virtual research environment in Europe to facilitate the work of biodiversity researchers and managers. LifeWatchGreece is one of these nodes where a portal was developed offering access to a suite of virtual laboratories and e-services.

**New information:**

Despite its strict definition in information technology, in practice "portal" is a fairly broad term that embraces many web architectures. In the biodiversity domain, the term "portal" is usually used to indicate either a web site that provides access to a single or an aggregation of data repositories (like: http://indiabiodiversity.org/, http://www.mountainbiodiversity.org/, http://data.freshwaterbiodiversity.eu), a web site that gathers information about various online biodiversity tools (like http://test-eubon.ebd.csic.es/, http://marine.lifewatch.eu/) or a web site that just gathers information and news about the biodiversity domain (like http://chm.moew.government.bg). LifeWatchGreece's portal takes the concept of a portal a step further. In strict IT terms, LifeWatchGreece's portal is partly a portal, partly a platform and partly an aggregator. It includes a number of biodiversity-related web tools integrated into a centrally-controlled software ecosystem. This ecosystem includes subsystems for access control, traffic monitoring, user notifications and web tool management. These subsystems are shared to all the web tools that have been integrated to the portal and thereby are part of this ecosystem. These web tools do not consist in external and completely independent web applications as it happens in most other portals. A quite obvious (to the user) indication of this is the Single-Sign-On (SSO) functionality for all tools and the common user interface wrapper that most of these tools use. Another example of a less obvious functionality is the common user profile that is shared and can be utilized by all tools (e.g user's timezone).

## Introduction

LifeWatch is an European e-infrastructure to support biodiversity and ecosystem research for societal benefits (Basset and Los 2012). It is composed of virtual laboratories supplied by the most advanced facilities to capture, standardize, integrate, analyze and model biodiversity data, and to elaborate and run possible scenarios of changes. LifeWatch is supported by the European Strategy Forum for Research Infrastructures initiative (ESFRI). Basset and Los 2012 It is distributed among 6 countries in Europe. Each country develops virtual laboratories over platforms developed locally with recommendations for interoperability (High Level Expert Group on Scientific Data 2010).

This paper reports on the development and operation of the LifeWatchGreece portal. This portal is the main software component of the e-infrastructure and constituted of a platform supporting the electronic services (e-Services) and the virtual laboratories (vLabs) of the RI. In this paper, we mainly focus on the overall portal description and architecture, leaving aside the details about each virtual laboratory or electronic service that is provided by the portal.Hernandez-Ernst et al. 2010

## Project description

### Design description

**Requirements**

The general requirements for the system's essential components were already set in the LifeWatchGreece project proposal documents as the following:

- e-Services (basic computing functionalities, not necessarily as web services): "*To pave the way for the development of complex virtual domains through a number of background e-Services [...]*."

"*The overarching objective [...] is to offer the basic e-Services to biodiversity data and information donors (contributors) and receptors (users). This component of the national infrastructure offers data and information services in order to address the questions what biodiversity exists and where. By basic services it is meant a system of software which can support: (a) the “machine-to-machine” interaction between the installed servers, (b) the submission and, (c) the delivery of biodiversity data and information. The e-Services are specifically designed to deliver also derived data and information, such as the calculation of a biodiversity index value or the value of an indicator, [...]*"

- vLabs (virtual laboratories): "*To develop a number of virtual labs where large scale science can be carried out at all possible levels of the biological organization from molecules to ecosystems*." "*The overarching objective [...] is to offer [...] virtual laboratories (vLabs) to the users of the infrastructure and especially to scientists. By vLabs it is meant a series of interrelated and interlinked modules or tools (software) which can facilitate users to address complex questions like why and how biodiversity occurs at a specific level of the biological organisation and on a specific spatio-temporal (or any other kind of observational) scale. The vLabs are based on the e-Services and allow for complex scientific hypotheses, such as the genetic or morphological identity of a species to be tested, and even managerial objectives, such as conservation and decision making, to be addressed through the resulting information. The tools may be integrated through specific APIs, to the LifeWatch Research Infrastructure or to any other e-Infrastructure, world wide*."

- Platform: "*[...] develop a common platform, [...] [where] different sectors/disciplines working with biodiversity aspects interact, [are] integrated and thus [act] in a harmonized way*." "*[...] a service electronic platform must be developed to provide the data providers with tools in order to make their data compliant to the “LifeWatch Reference Model” and through this service to make their “real” data available to any potential user, world wide*". Not many details and constraints were specified for the development of the platform, except about the development of data and metadata query/recovery functionality through ontology technology, and the necessity to follow the “*LifeWatch Reference Model*” (Hernandez-Ernst et al. 2010) and other documents prepared by the Preparation Phase of LifeWatch.

In summary, the e-Services are software components dedicated to biodiversity data mobilization and straightforward exploration. The vLabs are software components calling upon one or several e-Services to develop descriptive and predictive complex analyzes and models. The platform is a software component that proposes facilities to plug e-Services and vLabs under the same open web environment that ensures internal and external interoperability.

In the context of LifeWatch, vLabs from various national centres will be integrated into virtual research environments (VREs) dedicated to high level domains such as marine or freshwater biodiversity.

## Web location (URIs)

Homepage: https://portal.lifewatchgreece.eu

## Technical specification

Programming language: PHP, Java SE, J2EE, Javascript, Bash, C#

Interface language: HTML, CSS

## Usage rights

### Use license

Other

### IP rights notes

The code license for each web tool will be mentioned in its relevant paper.

## Implementation

### Implements specification

**Architecture**

The aforementioned web tools (e-Services and vLabs) share a common trait. They are targeting broadly the same user group and were going to be developed by the same team. So, a need for integration had become apparent early on, over the first stages of development. A user had to be able to navigate from one tool to the other easily. This is somehow apparent for web tools with a graphical interface but it also extends to the case of web services that communicate with each other in the background.

The guidelines to be followed in the development stage were not only dictated by the nature of the software itself but also by taking into account the available resources and technological background of the team. Security was one area where no compromises could be made. Easy integration of every new web tool was another important concern. Removing limitations in technologies that could be used to develop the web tools was vital, since that would allow any team member to easily develop new services and tools for the portal. Also, an extension of the platform concept to a portal concept became apparent while dealing with aspects related to graphical interface and client-side development (see also next paragraphs).

**Figure 1.**
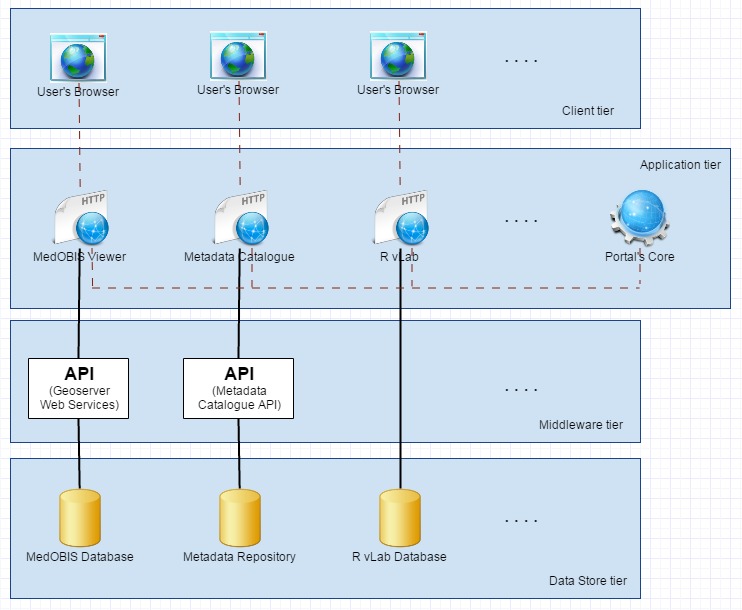
LifeWatchGreece layered architecture.

The LifeWatchGreece's portal is constituted by a number of web tools, which communicate with each other in various ways. A common authentication system and a level of consistency for the user interface of every web tool was implemented. However, each tool operates independently, so that removing any of the tools from the portal doesn't break anything in the others. In case a tool is using some services of another, the first should be able to adapt it's functionality when the second tool's services are not available (either due to maintenance and technical problems or due to its potential removal from the portal). The independence of each tool is partly achieved by using a layered architecture that loosely couples portal's components among themselves. The layered architecture of LifeWatchGreece portal is illustrated in Fig. 1

The base layer of this architecture is the Data Store layer where the data reside. It contains relational databases (e.g MySQL, PostgreSQL), triple stores (e.g Virtuoso), file repositories (e.g iRODS) etc. The concept of data is not limited to biodiversity-related datasets but is also extended to whatever data are used by the LifeWatchGreece portal, like logs, configuration, system state, activity records and any kind of data that is required for the operation of the integrated web tools.

The next layer is the Middleware layer where various APIs reside. These can be web-based APIs, or not, that are intended for use by portal's web tools, by third-party services or both. Custom APIs written in Java or PHP and APIs published by open source software (like Geoserver) can be found here. The development and use of APIs is of great importance in this effort to make more and more of LifeWatchGreece portal's services available to anyone who wants to develop new services and tools based on these.

The application layer contains all the web tools (e-services and virtual laboratories) that have been integrated to the LifeWatchGreece portal. Portal's functionality that is always available in every tool (e.g., the logout action), is implemented as a separate web application that, for the sake of this article, we will call Core App or Portal's core. This special application is vital to the functionality of the portal since it provides an endpoint to which every other web tool communicates with in order to enforce security and access control policies.

The last layer is the Client tier where user's browser resides. This is where the UI or some Javascript applications reside. Client tier is not less important since some applications are based on extensive communication between this tier and the Application or Middleware tier. A good example here is the case of MedOBIS viewer and its communication with WMS and WFS services of Geoserver.

**Interface**

**Figure 2.**
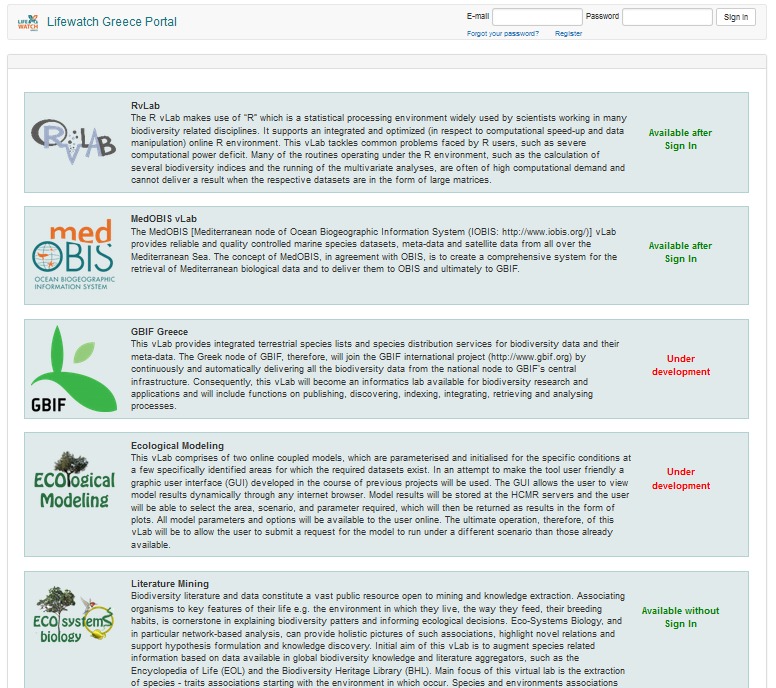
Portal's landing page.

The portal's landing page (Fig. 2) displays a list of web tools offered by LifeWatchGreece and the access policy for each tool. There are four such policies: “open”, “free”, “developing” and “controlled”. "Open" means that a tool can be accessed by anyone without having to register or login to LifeWatchGreece portal. "Free" means that anyone can access that tool but he first needs registration and login. "Developing" is used when a new tool is under development and so access to this tool is allowed only for a certain group of developers. Finally, "Controlled" means that access to this tool not only requires registration and logging in but, on the top of that, can be granted or revoked to specific users or groups of users. Whether a new portal user has by default access to this tool or needs to be granted access, depends on the configuration of this tool.

**e-services and vLabs**

Each of these web tools will be described in detail in its own separate paper. We present here a summary for those that are already part of LifeWatchGreece portal:

- R vLab: The R vLab makes use of “R” which is a statistical processing environment widely used by scientists working in many biodiversity related disciplines. It supports an integrated and optimized (in respect to computational speed-up and data manipulation) online R environment. This vLab tackles common problems faced by R users, such as severe computational power deficit. Many of the routines operating under the R environment, such as the calculation of several biodiversity indices and the running of the multivariate analyses, are often of high computational demand and cannot deliver a result when the respective datasets are in the form of large matrices.
- MedOBIS vLab: The MedOBIS [Mediterranean node of Ocean Biogeographic Information System (IOBIS: http://www.iobis.org/)] vLab provides reliable and quality controlled marine species datasets, meta-data and satellite data from all over the Mediterranean Sea. The concept of MedOBIS, in agreement with OBIS, is to create a comprehensive system for the retrieval of Mediterranean occurrence data and to deliver them to OBIS and ultimately to GBIF. Following a recent development in OBIS data model, it starts to deliver also associated environmental data.
- Ecological Modeling: This vLab is comprised of two online coupled models, which are parameterised and initialised for the specific conditions at a few specifically identified areas for which the required datasets exist. In an attempt to make the tool user friendly a graphic user interface (GUI) developed in the course of previous projects will be used. The GUI allows the user to view model results dynamically through any internet browser. Model results will be stored at the HCMR servers and the user will be able to select the area, scenario, and parameter required, which will then be returned as results in the form of plots. All model parameters and options will be available to the user online. The ultimate operation, therefore, of this vLab will be to allow the user to submit a request for the model to run under a different scenario than those already available.
- Literature Mining: Biodiversity literature and data constitute a vast public resource open to mining and knowledge extraction. Associating organisms to key features of their life e.g. the environment in which they live, the way they feed, their breeding habits, is cornerstone in explaining biodiversity patterns and informing ecological decisions. Eco-Systems Biology, and in particular network-based analysis, can provide holistic pictures of such associations, highlight novel relations and support hypothesis formulation and knowledge discovery. Initial aim of this vLab is to augment species related information based on data available in global biodiversity knowledge and literature aggregators, such as the Encyclopedia of Life (EOL) and the Biodiversity Heritage Library (BHL). Main focus of this virtual lab is the extraction of species - traits associations starting with the environment in which occur. Species and environments associations will be extracted by mining relevant text field clauses of: a) in-house LifeWatchGreece data, b) the EOL and the BHL text collection. Also, interactive web-based visualizations will be developed to summarise the extracted species - environments association and support data exploration and landscape ecology studies.
- Data Services: Data Services provide the users with tools in order to: a) publish their datasets and make them available to the community by providing information that allows a user to locate and access the resource and its curator/creator, b) import their datasets to the Lifewatch Greece Infrastructure, c) search about datasets of interest by providing an efficient way of querying semantic networks, d) annotate species using morphological traits, e) perform biodiversity data and information quality improvement. The schema of the data that is provided by the users is mapped to the semantic model of the LWI and the data is transformed to LWI format before it is stored to the Infrastructure. The semantic model is based on CIDOC CRM, CRM dig, CRM geo, CRM sci and MarineTLO.
- Micro-CT vLab: Micro-tomography (micro-computed tomography or microCT) is a method of non-destructive 3D x-ray microscopy, which allows the users to create 3D models of objects from a series of x-ray projection images, similar to the conventional clinical computer tomography. The MicroCT Service will offer a collection of virtual galleries of taxa which will be displayed and disseminated through a web-based framework, and will allow the user to manipulate the 3D models through a series of online tools or to download the datasets for local manipulations.
- Genetics services: The online Services of the Genetics working group are focused on 16S Metagenomics, which involve specific 16S ribosomal marker gene amplification by PCR. The 16S analysis is the most common approach for biodiversity studies. The Service focuses on taxonomic analysis from data derived from 454 Roche as well as Illumina sequencing technologies and provides the relevant software to allow analysis for both sequencing technologies. Efficient noise removal for 454 data is accomplished using the AmpliconNoise (http://qiime.org/scripts/ampliconnoise.html) pipeline and the taxonomic assignment for both de-noised 454 and Illumina data is achieved via QIIME (http://qiime.org/index.html).
- Taxon Information System (TIS) Services: This Service acts as the taxonomic backbone of the infrastructure: a taxonomic identity code (ID) is assigned to all the taxa registered in the TIS. All the information (e.g. systematic classification, taxonomic description, functional trait information, geographical distribution, registered material in the systematic collections of the Museums, etc.), which is relevant to a taxon can be assigned to its ID and through this to any other kind of information linked to that. The TIS Service is directly based on those developed in Flanders Marine Institute (VLIZ), which have been developed in the course of several EU and international projects and initiatives, such as MarBEF (Marine Biodiversity and Ecosystem Functioning), PESI (Pan-European Species directories Infrastructure), WoRMS (World Register of Marine Species), and CoL (Catalogue of Life). The core of this service is dedicated to the list of species in Greece, called GTIS for Greek Taxonomic Information Service,
- Mobile Applications: Many of LifeWatchGreece web applications are also available as mobile applications. Currently there is a main application called "LifeWatch Greece" Mobile app that consists of 4 sub-applications, two of them available without login and the other two by using LifeWatchGreece portal credentials. Data from different Citizen Science projects such as Comber and CIGESMED can be seen in Citizen Science sub application. MicroCTvlab, RvLAB and MedOBIS are sub applications where the user has the ability to see most of the options that are available through the LifeWatchGreece portal. The "LifeWatch Greece" Mobile app is available for free at the Google Play Store.

**Figure 3.**
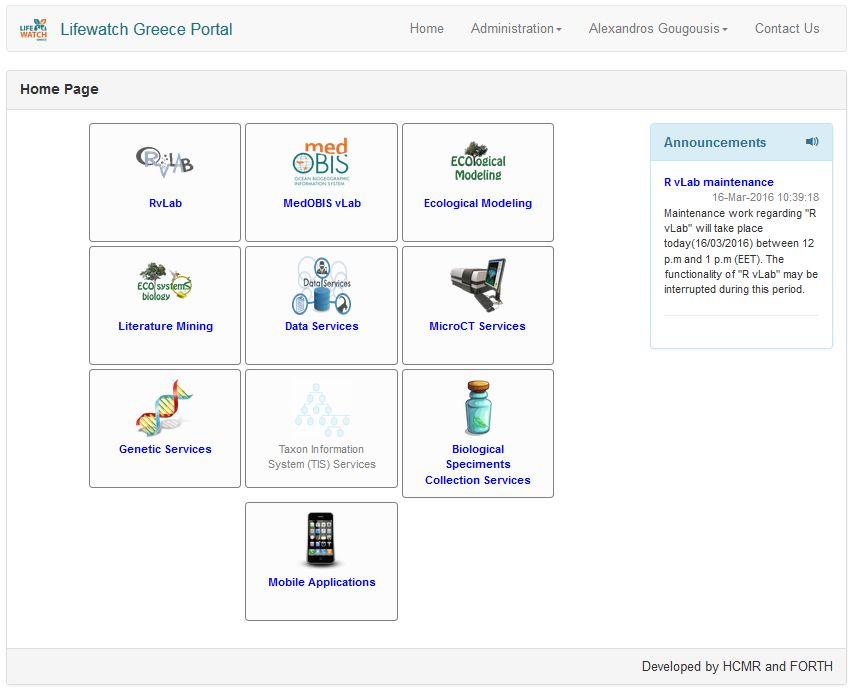
Portal's home page.

In Fig. 3, the portal's Home Page is displayed. This is the first page a user lands on after logging in. In the center of the page there is list of buttons, one for every application that has been integrated or is being integrated to the portal. Tools that are not accessible (e.g in the "developing" state) by the user are faded. Important announcements related to the portal as a whole or a specific tool are displayed in the right part of the screen. A menu is available on the top bar, where administrative functions are available for users with administrative rights and some basic options for the rest of users (like editing his/her profile).

Information that belongs to the user profile can be made available to all tools in order to be utilized appropriately. For example, user's timezone is being utilized by R vLab , in order to display correctly the dates and times when the user submitted specific jobs.

**Figure 4.**
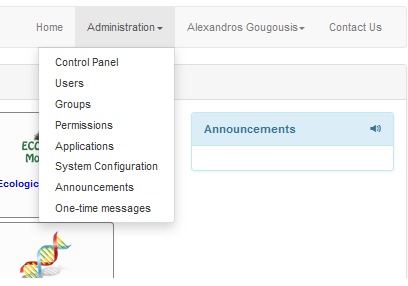
Administration menu.

The administrative functions (Fig. 4) are mainly dealing with issues related to access control, notification messages and announcements, and system configuration. The portal's administrator can manage registered users or manually register a new user. In case of suspicious user activity, the administrator can deactivate a user account and reactivate it later if needed. Permissions can be granted and revoked on a per-user or per-group basis. Users can be assigned to groups, new groups or permissions can be created in order to be used by specific (or all) web tools. This architecture allows building functionality in a specific web tool but managing the access to this functionality from a central point and for every portal user or group.

**Figure 5.**
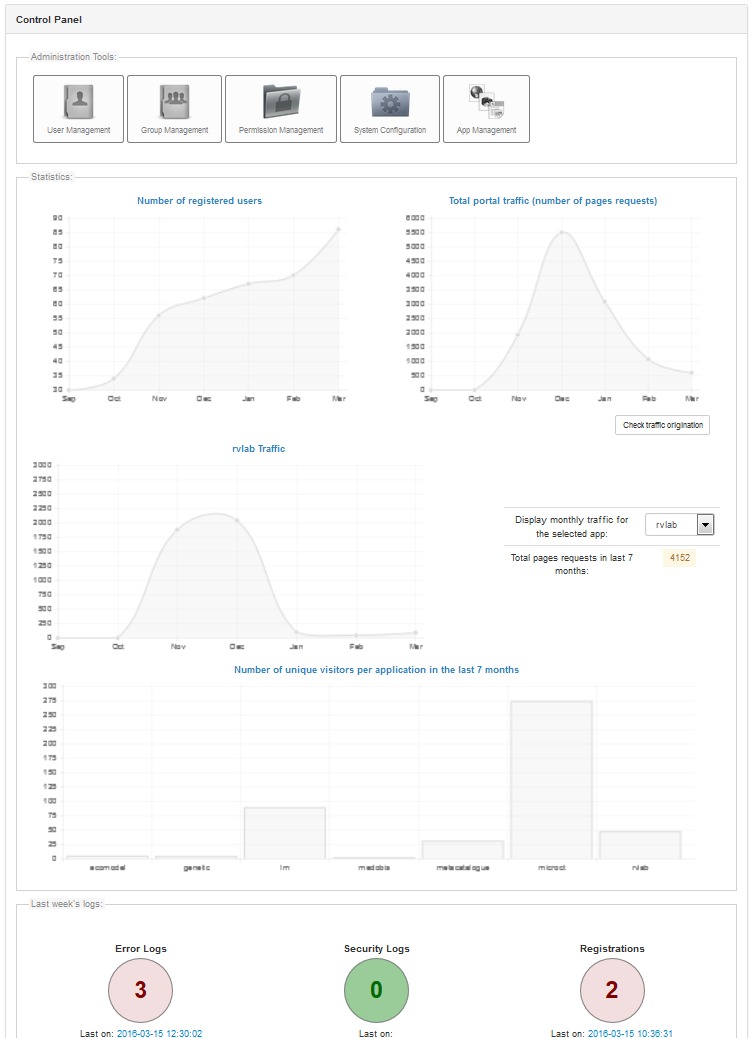
Control panel.

A control panel (Fig. 5) has been built in order to have a quick overview on issues regarding security, traffic, online users, etc. Any sign of misuse should be evaluated, further investigated and measures should be taken if required. For example, if a sudden increase in registrations is noticed by the administrator, that might means a security hole exists in the registration. An unusual level of traffic may be a sign of an attack etc.

**Figure 6.**
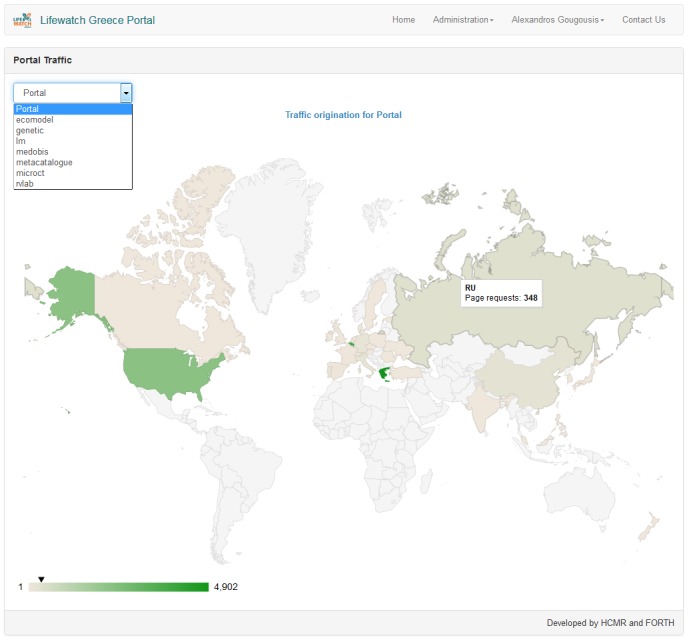
Portal traffic statistics (volume and user origin).

Traffic statistics can also derived by logs. Information about the operating system and the browser as well as the user's origin (e.g country) can be extracted. A graphical representation (Fig. 6) of the locations that generate this traffic and the traffic volume that corresponds to each location is also availableFig. 6. This is just an estimation since identifying the real traffic is not always that simple. AJAX requests, requests coming from institute's intranet and traffic that comes from bots and web crawlers (identified on best-effort basis) has been excluded. Resolving IP addresses to countries takes place utilizing http://ipinfo.io/ services.

Another useful feature is that we can maintain the software version for the mobile version of each web tool that has been included in the LifeWatchGreece mobile application. By updating the version of a specific tool, the mobile application can inform the user about the need for updating the application without blocking access to the other tools.

**Access Control**

Portal's access control scheme works in two levels. The first level is the access policy that can be selected for each web tool. The "open" policy puts no restrictions at all. The "free" policy requires user authentication (register and log in before being able to access the web tool). Those two policies work only on the first level. The other two policies, "developing" and "controlled", which also require authentication, work on the second level, too. They are based on predefined permissions for each web tool that can be manually or automatically (based on rules) granted to users or groups of users by administrators. Moreover, custom permissions can be defined and granted for each web tool (on a portal-level), which can then be used by the web tool developer to build a more fine-grained access control scheme for the specific web tool.

Most of the tools provided by the LifeWatchGreece portal are available only after registration (because logging in is required). Controlled access to these web tools was necessary for many reasons. First of all, for security reasons. We needed a way to identify the source of traffic in case a service is being abused. Secondly, there are tools that should provide a minimum Quality of Service (QoS) in order to be useful. For example, jobs submitted in R vLab should be executed in a sensible time. Controlling this QoS requires the control of incoming traffic, which is directly affected by the number of users served by the corresponded tool. Getting a step further, we should not forget that logging in allows us to offer some personalized services or working environment. For example, R vLab will not able to function without user identification. Lastly, as we already have mentioned, traffic logs help the administrator to form a clear picture about the user's distribution and popularity of each web tool. Moreover, it allows to make predictions about the volume of the traffic that a single user creates and about the future traffic that we will need to serve, something that needs to be taken into account in the mid- to long- term design of our services.

### Audience

By their objects (genes, species, ecosystems), their nature and above all to the degree of their complexity, the biodiversity RIs need to target a large variety of audiences. However, the diversity of data and tools proposed by this type of web-based software complicates their communication to the potential end-users as well as their acceptance and endorsement as a tool for daily use by researchers and experts. It is thus important to clearly segment the functionalities along with the targeted public in order to increase the understanding and the visibility of vLabs and applications.

One way to achieve that communication goal is to refer to the "data to wisdom hierarchy" (Zelený 1987, Ackoff 1989) by associating data and tools to one of its level (data, information, knowledge, wisdom) and indicating the type of users from specialist researchers to public at large.

As a general rule the data and information levels are more targeting specialists while knowledge and wisdom levels are more targeting policy and decision makers, and public at large. The same stands for the tools, e-services and vLabs. For instance, in the MedOBIS vLab, the use of data requires a critical approach to filter out doubtful pieces of data, which is only manageable by researchers and students under guidance. The produced point maps cannot be correctly interpreted without a good knowledge of how chorology is studied. It is the same for the rVlab that mainly transforms data into information through sophisticated statistical analysis that obviously can be handled only by specialists. At the opposite, the GTIS vLab that provides lists of species in Greece falls also within the competence of biodiversity managers and public at large, including school children. Likewise, the mobile phone application linked to the Micro-CT vlab can be used by the public at large, even if the purposes may be rather different.

The difference of targeted public groups also impacts the design of the user interfaces that may be quite simple for researchers (who may even prefer that) while they must be quite sophisticated for the public at large. In any case, the interfaces must be adapted to the effective use of both data and tools.

## Additional information

### Challenges met and Solutions

One of the greatest challenges faced during the development phase of the LifeWatchGreece RI was the diversity of developers' background. Web tools were being developed not only by software developers but also by biologists, environmentalists, physicists etc. with various levels of programming background and skills. Moreover, not all developers were aware the basics of latest technologies and standards, like HTML5. The development of a portal and the integration of all these web tools to a common platform requires high degree of coordination. The chosen layered portal architecture was only the first step. As a second step, basic guidelines about structuring the HTML pages were given to all developers. From there on, different solutions were put forward on a case by case basis. When the web tool was built as a web service/API, not much needed to be done. In some cases, where the development of a web tool was in an advanced stage, manual modifications had to be made by software developers. In other cases, the web tool was refactored so that adding functionality could be done by giving guidelines to the developers and showing them how to imitate the coding patterns that had been used. The last resort, for some remaining cases, was to utilize iframes. However, we tried to minimize the use of iframes and be extremely cautious, due to security concerns.

### Future Improvements and Developments

In order to encourage the development of third-party services based on LifeWatchGreece's services, the architecture of portal's web tools should be revisited. A more API-oriented approach fits better to the purpose of these tools. This is also a lesson given by our effort to develop and maintain a mobile version for some tools. A few of portal's web tools have already been built on that basis but not all. Some of them follow the MVC architecture that makes a first separation between the business logic and the user interface but this proved not to be enough. A Service Oriented Architecture (SOA) or a REST-based Oriented Architecture (ROA) seems more appropriate for LifeWatchGreece portal. Separating vLabs into completely independent web applications (front-end application and back-end services) increases the overall architecture's flexibility significantly. Individual services can be scaled independently of each other and can also be maintained by different teams or team members. Though portal's particularities should be carefully taken into account, market trends over the last years show clearly that the community moves slowly but steadily from SOA to ROA (Google 2016). Facebook, Youtube, Google Maps and Twitter has long ago turned to JSON-based services. In any case, such improvements on the architecture will also make easier to combine functionality of several web tools and provide richer services to the community.

Another important issue is the development of portal's control panel. In its current state, the logs that are being monitored by the control panel concern only portal's core functionality. Automatic loading of crucial logs from each web tool to the central control panel would greatly help spotting potential problems on time. Moreover, a finer level of statistics can be produced by separating traffic for each web tool or displaying graphs about the O.S. and browser generally or by web tool.

In addition, the AAI (Authentication Authorization Identification) part can be further improved. Though portal uses a Single-Sing-On (SSO) system, which allows a user to access many web tools with a single registration and login, there is one more pair of credentials that needs to be kept by the user. Eliminating the need for registration by adding support to third-party authentication systems is widely accepted in the scientific community and, therefore, it can be an important step. As an example, authentication through ORCID is already an ongoing work. Furthermore, supporting third-party authentication systems coming from widely accepted systems like social media web applications (e.g LinkedIn, Facebook etc.) can also be a topic for further discussion.
